# Current trends on antifungal prophylaxis in solid organ transplantation: a study from ESCMID-EFISG, ESCMID-ESGICH, SITA, and SEIMC-GESITRA-IC

**DOI:** 10.1007/s15010-025-02575-z

**Published:** 2025-07-04

**Authors:** Jon Salmanton-García, Alessandro Giacinta, Maddalena Giannella, Antonio Vena, Patricia Muñoz, Oliver A. Cornely, Maricela Valerio

**Affiliations:** 1 Institute of Translational Research, Cologne Excellence Cluster On Cellular Stress Responses in Aging-Associated Diseases (CECAD), Faculty of Medicine, University of Cologne, Herderstraße 52, 50931 Cologne, Germany; 2 University Hospital Cologne, Cologne, Germany; 3 Department I of Internal Medicine, Center for Integrated Oncology Aachen Bonn Cologne Duesseldorf (CIO ABCD) and Excellence Center for Medical Mycology (ECMM), Faculty of Medicine, University of Cologne, University Hospital Cologne, Cologne, Germany; 4 German Centre for Infection Research (DZIF), Partner Site Bonn-Cologne, Cologne, Germany; 5 Department of Clinical Microbiology and Infectious Diseases, Hospital General Universitario Gregorio Marañón, Madrid, Spain; 6 Division of Infectious Diseases, Department of Medicine, University of Udine, Udine, Italy; 7 Infectious Diseases Unit, IRCCS-Sant’Orsola Polyclinic, Bologna, Italy; 8 Department of Medical and Surgical Sciences, University of Bologna, Bologna, Italy; 9 Department of Health Sciences (DISSAL), University of Genoa, Genoa, Italy; 10 Clinica Malattie Infettive, IRCCS San Martino Polyclinic Hospital, Genoa, Italy; 11 Instituto de Investigación Sanitaria Gregorio Marañón, Madrid, Spain; 12 Department of Medicine, School of Medicine, Universidad Complutense de Madrid, Madrid, Spain; 13 Centro de Investigación Biomédica en Red de Enfermedades Respiratorias (CIBERES), Instituto de Salud Carlos III, Madrid, Spain; 14 Clinical Trials Centre Cologne (ZKS Köln), Faculty of Medicine, University of Cologne, Cologne, Germany

**Keywords:** Invasive fungal diseases, Solid organ transplant, Antifungal, Prophylaxis, Fungal epidemiology, Biomarker, Breakthrough IFD

## Abstract

**Introduction:**

Invasive fungal diseases (IFD) present serious risks to solid organ transplant recipients, particularly in the first 180 days post-transplant. Existing European and US guidelines offer limited evidence, prompting a shift away from universal prophylaxis due to adverse effects, drug-interactions, and costs. This study investigates antifungal prophylaxis practices in transplant centers to guide IFD management.

**Methods:**

From May 2023 to May 2024, tertiary care institutions completed an online survey on antifungal prophylaxis post-transplant. Data included transplant volumes, IFD incidence by pathogen, and prophylactic strategies.

**Results:**

Responses from 64 centers in 32 countries, mainly in Europe, highlighted kidney and liver as the most common transplants. Prophylaxis was universal in lung transplants and common in liver, bowel, and heart transplants, often triggered by reintervention or *Candida* spp. colonization. Preferred agents included liposomal amphotericin B and fluconazole.

**Conclusions:**

This global survey reveals substantial variation in antifungal prophylaxis practices among solid organ transplant centers, driven by a lack of standardized, evidence-based guidelines. Our findings underscore the urgent need for harmonized recommendations that reflect evolving fungal epidemiology, improved diagnostics, and new antifungal agents.

**Supplementary Information:**

The online version contains supplementary material available at 10.1007/s15010-025-02575-z.

## Introduction

Invasive fungal diseases (IFD) are opportunistic diseases in solid organ transplant recipients. The predominant fungal pathogens, *Aspergillus* spp. and *Candida* spp., are most frequently observed within the initial 180 days post-transplantation [1]. Specifically, transplant recipients of bowel and lung exhibit the highest risk for secondary IFD, followed sequentially by recipients of liver, heart, pancreas, and kidney transplants [1]. Despite recommendations from Europe and United States do exist, these are sparse and founded on limited evidence [2–4]. Furthermore, the rapidly changing fungal epidemiology—driven by post-respiratory viral infection IFD, the emergence of resistant fungal species, the increasing use of ventricular assist devices and extracorporeal membrane oxygenation (ECMO) and the new antifungal available [5]—challenges the applicability of such documents developed in 2014 and 2019, respectively.

In parallel, prophylactic strategies are concurrently undergoing transformation. Universal prophylaxis is becoming less frequent due to the associated risks, including the emergence of IFD caused by rare fungi (i.e., Mucorales or *Lomentospora*/*Scedosporium* spp.), adverse effects, drug-drug interactions (notably with immunosuppressants), and increased costs [5]. Nowadays, fungal biomarkers can provide more precise guidance for preemptive therapy but its role in antifungal prophylaxis is not so clear. Moreover, the introduction of new antifungal agents, which target various fungal cellular components, offer broader antifungal spectra, and have improved interaction profiles with drugs used for comorbid conditions, has significantly altered the therapeutic landscape. However, there remains a lack of high-quality evidence from clinical trials on the use of these new antifungal agents as prophylaxis in solid organ transplant recipients, with the current knowledge predominantly originating from audits, case reports and anecdotal clinical experience.

This study aims to delineate the current antifungal prophylaxis patterns in institutions managing solid organ transplant patients and establish a foundation for evidence-based guidelines on antifungal prophylaxis management in this high-risk patient population.

## Methods

Between May 2023 and May 2024, tertiary care institutions were invited to participate in a predesigned questionnaire addressing key aspects of antifungal prophylaxis is following solid organ transplantation. The questionnaire covered the (a) number of transplantations per organ (bowel, heart, kidney, liver, lung, and pancreas) performed in 2022 at each institution, (b) number of diagnosed IFD in organ transplant recipients, categorized by pathogen within the previous 10 years to allow an assessment of longer-term trends, and (c) antifungal prophylaxis strategies, including initiation triggers, preferred AF, and duration of administration. Data were collected using an online electronic case report form (eCRF) hosted at https://www.clinicalsurveys.net/uc/AFprophylaxisSOT/ (EFS, TIVIAN GmbH, Cologne, Germany). Before analysis, the data underwent formal monitoring to ensure completeness and consistency and to avoid duplicate participation of authors from the same institutions. For duplicated responses, the most complete entry from each center was retained. If multiple complete responses were received, we verified with respondents for the most updated and institutionally endorsed submission.

Invitations were sent to members of scientific organizations, including the European Society of Clinical Microbiology and Infectious Diseases (ESCMID), specifically the Fungal Study Group (EFISG) and the Study Group for Infections in Compromised Hosts (ESGICH), the Study Group on Infection in Transplantation and Immunocompromised Hosts (GESITRA-IC) of the Spanish Society of Infectious Diseases and Clinical Microbiology (SEIMC), and the Italian Society of Anti-Infective Therapy (SITA). Additionally, calls for participation were posted online on LinkedIn^®^ and Twitter/X^®^ social media platforms.

The data presentation includes frequencies and percentages, organized in contingency tables and figures. Continuous variables are presented with their median, interquartile range (IQR) and absolute range. No statistical comparisons were made due to the exploratory nature of this analysis. Data were processed using SPSS v27.0 (SPSS, IBM Corp., Chicago, IL, United States).

## Results

### Demographic characteristics

**Fig. 1:**
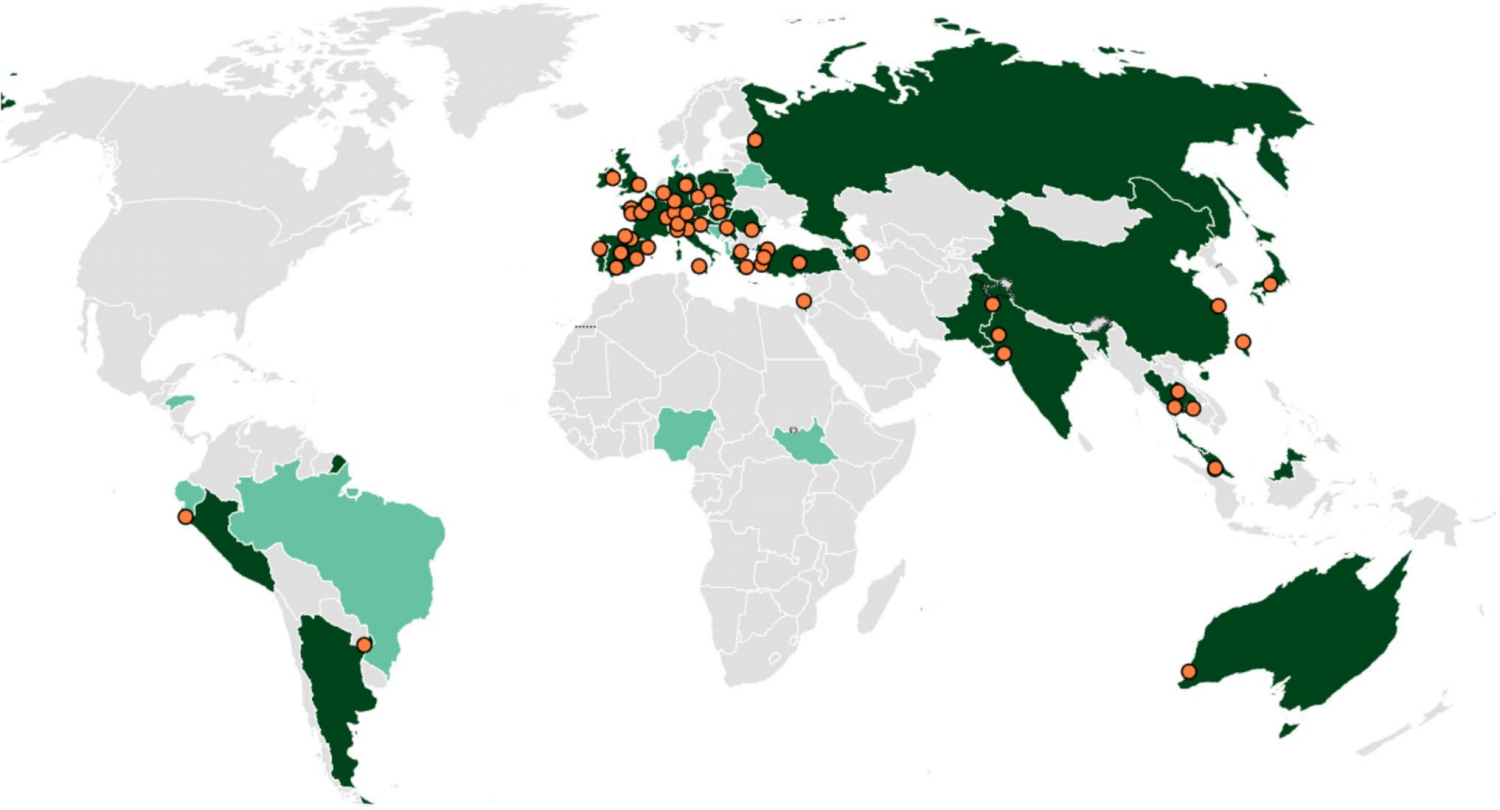
Geographical distribution of participating transplantation units. Countries colored in grey have no participating institutions or have only institutions with incomplete responses. Light green indicates countries with at least one participating institution that does not perform transplants. Dark green indicates countries with at least one participating institution that does performs transplants. Orange points mark the locations of participating transplant units; if two units are in the same location, they are represented by a single point. Participating units belong to Spain (11/64, 17.2%), France (8/64, 12.5%), Italy (5/64, 7.8%), Turkey (4/64, 6.3%), Germany and Ireland (3/64, 4.7% each), Greece, India, Switzerland, and Thailand (2/64, 31.1% each), and Argentina, Australia, Austria, Azerbaijan, China, Croatia, Czechia, Hungary, Israel, Japan, Malaysia, Pakistan, Peru, Poland, Portugal, Romania, Russia, Serbia, Singapore, Slovakia, Taiwan, and United Kingdom (1/64, 1.6% each)

**Fig. 2:**
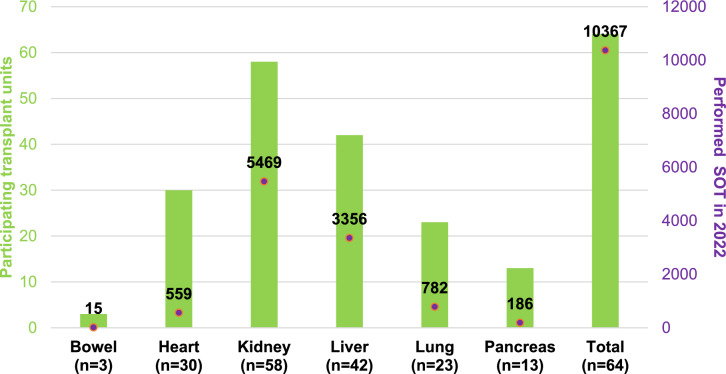
Participating institutions by organ and the number of transplantations performed for each organ in 2022

A total of 182 responses from 162 medical institutions worldwide were collected in the online survey. After excluding invalid responses—66/182 (36.3%) incomplete, 29/182 (15.9%) from non-transplant institutions, and 23/182 (12.6%) duplicated entries from same institutions—we analyzed data from 64/182 valid responses (35.2%) across 32 countries, mostly in Europe. Spain had the highest response rate (11/64, 17.2%), followed by France (8/64, 12.5%) and Italy (5/64, 7.8%) (Fig. 1). Most participants were from institutions performing kidney transplants (58/64, 90.6%), followed by those involved in liver transplants (42/64, 65.6%). Only 14/64 institutions (21.9%) performed transplants of a single organ, primarily kidneys (11/64, 17.2%). In the previous year, these institutions reported performing 7 bowel, 559 heart, 5469 kidney, 3356 liver, 782 lung, and 186 pancreas transplants (Fig. 2).

### Air quality

Air sampling was an established practice in 24.2% of the institutions (15/62), with the highest rates in lung (6/23, 26.1%) and heart (7/29, 24.1%) transplant units. High-efficiency particulate air (HEPA) filters were used in 46.9% of institutions (30/64), varying by organ type, with 60.0% of lung units (14/23) and 50.0% of heart units (15/30) using these filters (Table 1).

**Table 1:** Air sampling, utilization of high-efficiency particulate air (HEPA) filters, implementation of antifungal prophylaxis, and initiation triggers for antifungal prophylaxis depending on the transplanted organ

	All sites		Bowel		Heart		Kidney		Liver		Lung		Pancreas	
	n	%	n	%	n	%	n	%	n	%	n	%	n	%
Air sampling	15/64	23.4	0/3	0.0	7/29	24.1	12/58	20.7	9/41	22.0	6/23	26.1	1/13	7.7
HEPA filters														
Always	7/64	10.9	0/3	0.0	4/29	13.8	7/58	12.1	7/41	17.1	3/23	13.0	1/13	7.7
Post-operatively	11/64	17.2	1/3	33.3	11/29	37.9	11/58	19.0	11/41	26.8	11/23	47.8	3/13	23.1
Depending on the organ	12/64	18.8	–	–	–	–	–	–	–	–	–	–	–	–
No HEPA	34/64	53.1	2/3	66.7	14/29	48.3	31/58	53.4	24/41	58.5	9/23	39.1	9/13	69.2
Antifungal prophylaxis performed	56/64	87.5	2/3	66.7	18/30	60.0	20/58	34.5	36/42	85.7	23/23	100.0	6/13	46.2
Universal prophylaxis	42/56	75.0	½	50.0	9/18	50.0	4/20	20.0	3/36	8.3	13/23	56.5	NC	NC
Triggers for targeted antifungal prophylaxis														
Re-transplantation	39/56	69.6			7/18	38.9			33/36	91.7				
Fulminant hepatic failure	31/56	55.4							30/36	83.3				
* Candida* spp. colonization	30/56	53.6	0/2	0.0			16/20	80.0	20/36	55.6			NC	NC
Re-intervention	29/56	51.8			5/18	27.8			24/36	66.7	1/23	4.3	NC	NC
Donor colonization	28/56	50.0	0/2	0.0	9/18	50.0	12/20	60.0	14/36	38.9	10/23	43.5	NC	NC
Hemodialysis	25/56	44.6	0/2	0.0	7/18	38.9	10/20	50.0	16/36	44.4			NC	NC
Transplant rejection	24/56	42.9	1/2	50.0	7/18	38.9	8/20	40.0	17/36	47.2	5/23	21.7	NC	NC
CVVH	24/56	42.9	0/2	0.0	9/18	50.0	9/20	45.0	17/36	47.2	2/23	8.7	NC	NC
Parenteral nutrition	23/56	41.1	0/2	0.0	7/18	38.9	13/20	65.0	14/36	38.9	0/23	0.0	NC	NC
Multiple transfusions	22/56	39.3							19/36	52.8				
Prolonged antimicrobials	21/56	37.5	0/2	0.0	5/18	27.8	13/20	65.0	14/36	38.9	0/23	0.0	NC	NC
CMV infection/reactivation	21/56	37.5	0/2	0.0	7/18	38.9	11/20	55.0	9/36	25.0	3/23	13.0	NC	NC
Prolonged MV/ICU	21/56	37.5			4/18	22.2			10/36	27.8	3/23	13.0	NC	NC
ATG	20/56	35.7			6/18	33.3	10/20	50.0	8/36	22.2	2/23	8.7		
ECMO	18/56	32.1			9/18	50.0			7/36	19.4	3/23	13.0	NC	NC
High fungal spore concentration	12/56	21.4	0/2	0.0	6/18	33.3	7/20	35.0	7/36	19.4	3/23	13.0	NC	NC
Delayed sternal closure	10/56	17.9			8/18	44.4					4/23	17.4		
Ventricular assist devices	8/56	14.3			4/18	22.2								

Denominator corresponds to the number of transplantation units performing prophylaxis for the respective organ. Numerators can be superadditive

*AF* antifungal, *ATG* Anti-thymocyte globulin, *CMV* cytomegalovirus, *CVVH* continuous veno-venous hemofiltration, *ECMO* extracorporeal membrane oxygenation, *HEPA* high-efficiency particulate air (filters), *ICU* intensive care unit, *MV* mechanical ventilation, *NC* not collected

### Antifungal prophylaxis: administration patterns

Administration of antifungal prophylaxis following lung transplantation was reported across all analyzed centers (23/23, 100%), with liver (36/42, 85.7%), bowel (2/3, 66.7%), and heart transplants (18/30, 60.0%) showing lower implementation rates. Universal prophylaxis was implemented in 50.0% of bowel transplants (1/2), 50.0% of heart transplants (9/18), 20.0% of kidney transplants (4/20), 8.3% of liver transplants (3/36), and 56.5% of lung transplants (13/23). Triggers for targeted bowel transplant prophylaxis included reintervention, organ retransplantation, or organ rejection (1/2, 50.0% each). In heart transplants, continuous veno-venous hemofiltration (CVVH), donor fungal colonization, or ECMO administration (9/18, 50.0% each) were common reasons for initiating prophylaxis. In kidney transplants, prophylaxis was often prompted by *Candida* spp. colonization (16/20, 80.0%), while in liver transplants due to organ retransplantation (33/36, 91.7%) and organ failure (30/36, 83.3%). For lung transplants, donor colonization (10/23, 43.5%) was the primary trigger. However, there was considerable variability in prophylaxis initiating reason across centers (Table 1).

### Antifungal prophylaxis: preferred antifungal and duration

For bowel transplants, preferred antifungal included liposomal amphotericin B or caspofungin (1/2, 50.0% each). For heart transplant prophylaxis the primarily used antifungal were echinocandins like anidulafungin (5/18, 27.8%) or caspofungin (4/18, 22.2%), and fluconazole (4/18, 22.2%). For kidney transplant prophylaxis the most commonly used was fluconazole (10/20, 50.0%). For liver transplant recipients the preferred antifungal for prophylaxis were caspofungin (15/36, 41.7%), fluconazole (14/36, 38.9%), or anidulafungin (11/36, 30.6%). For lung transplant prophylaxis the most common antifungal was inhaled amphotericin B (14/23, 60.9%) followed by voriconazole (12/23, 52.2%) as the second most common choice. And finally, for pancreas transplants, fluconazole was preferred (4/6, 66.7%) (Table 2).

**Table 2:**
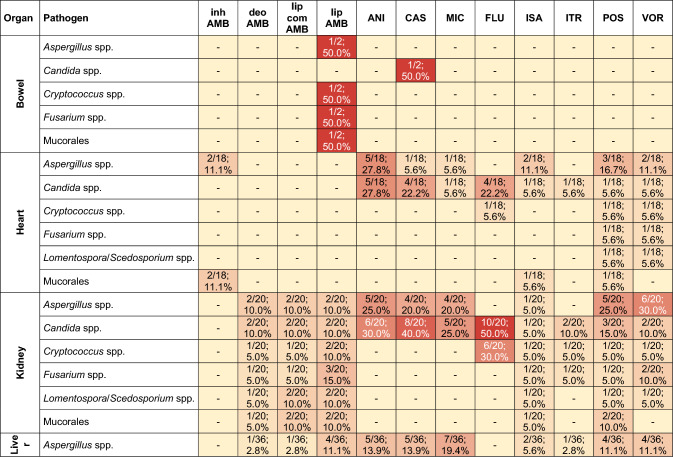
Cross-reference table of administered antifungal drugs for prophylactic use, categorized by pathogen and organ

Denominator corresponds to the number of transplantation units performing prophylaxis for the respective organ. Numerators can be superadditive

Cell colors span from pale yellow to deep red. The intensity of the color indicates the proportionately higher proportional of the respective antifungal in the corresponding organ-pathogen prophylaxis

*ANI* anidulafungi, *CAS* caspofungin, *deo AMB* deoxycholate AMB, *FLU* fluconazole, *inh AMB* inhaled AMB, *ISA* isavuconazole, *ITR* itraconazole, *lip AMB* liposomal AMB, *lip com AMB* lipid complex AMB, *MIC* micafungin, *POS* posaconazole, *VOR* voriconazole, *spp.* species

Regarding the duration of prophylaxis for at-risk patients, a limited number of centers had a consistent strategy. Prophylaxis duration was frequently described as up to 28 days post-transplantation for bowel, heart, kidney, liver, and pancreas transplants, regardless of the pathogen. In lung transplantation, 78.3% (18/23) of centers reported a prophylaxis duration longer than 28 days, especially for *Aspergillus* spp. (Supplementary Table 1).

### Breakthrough IFD

**Fig. 3:**
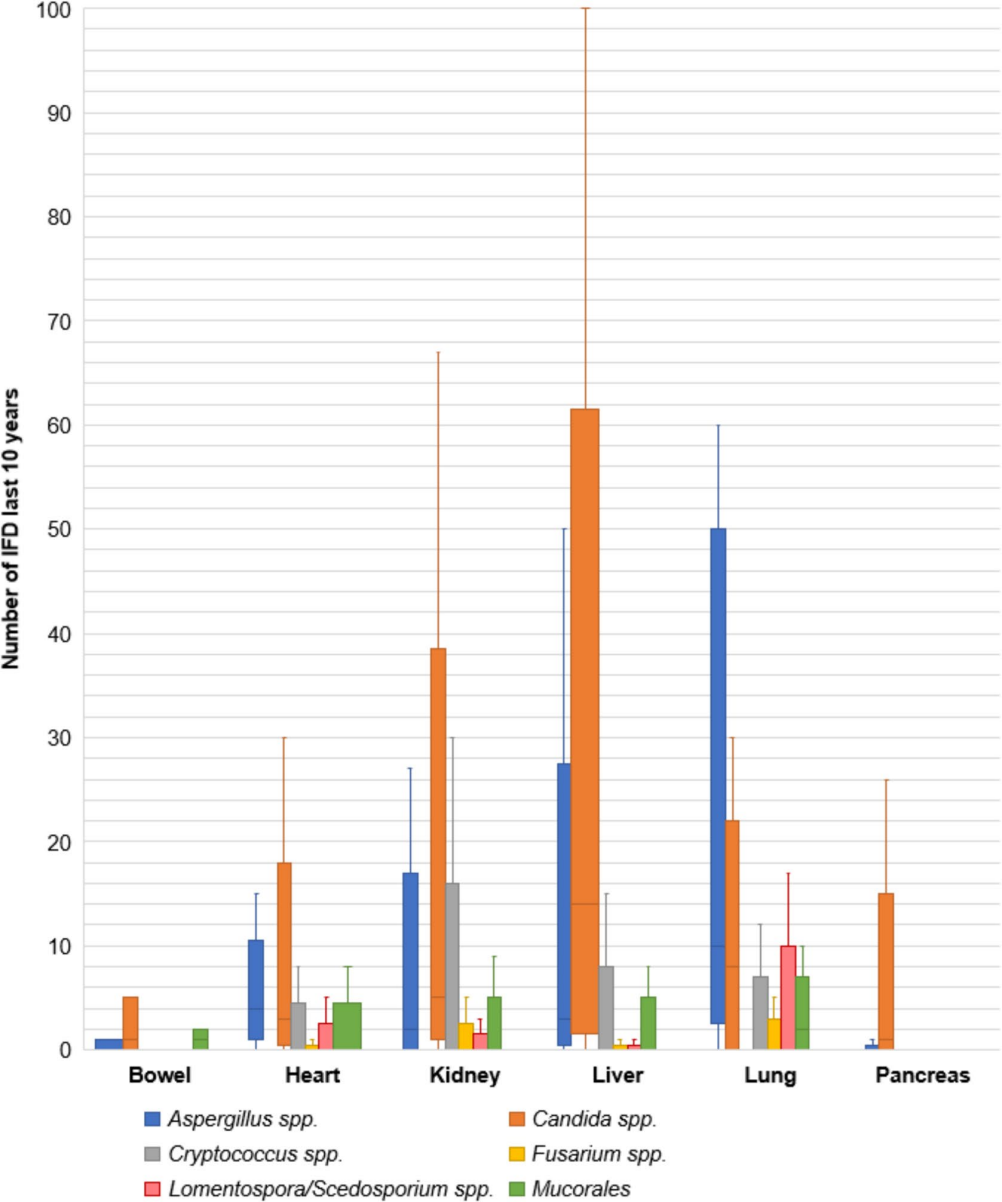
Reported post-transplant invasive fungal diseases over the last 10 years, categorized by pathogen and organ. *IFD* invasive fungal disease, *spp.* species

Responses regarding IFD over the past 10 years in each centre immediately before the participation in the survey, numbers varied based on the type of transplant and the specific pathogen involved. Estimated breakthrough IFD due to *Candida* spp. notably impacted liver transplant recipients, with a median of 15 cases (IQR 3–24, range 0–450). In lung transplants a median of 7.5 cases (IQR 0–14, range 0–30) due to *Candida* spp. was reported, while in kidney transplants a median of 5 cases (IQR 2–10, range 0–150). As for *Aspergillus* spp., IFD were most commonly observed in lung transplants, with a median of 10 cases (IQR 5–40, range 0–60). Heart transplant units handled a median of 4 cases (IQR 2–6, range 0–15) of *Aspergillus* spp. IFD. Mucorales-driven IFD were reported in lung transplants, with a median of 1.5 cases (IQR 0–4, range 0–10) (Fig. 3).

Nearly three-quarters of the sites (46/64, 71.9%) reported to have a dedicated team of infection specialists serving the transplant units.

## Discussion

This survey highlights considerable variability in antifungal prophylaxis practices and environmental controls across transplant centers internationally. Although nearly half of centers utilize HEPA filtration, its implementation is inconsistent and often limited to specific organ transplant units, such as lung and heart. The widespread use of prophylaxis in lung transplant recipients contrasts with the more selective application in other organ groups, reflecting divergent perceptions of IFD risk and institutional protocols. The preferred antifungal agents and treatment durations also differ markedly, with extended prophylaxis primarily reserved for lung transplants—likely due to their uniquely high vulnerability to IFD. These findings underscore the absence of universally adopted guidelines and emphasize the need for standardized, evidence-based strategies that account for organ-specific risks and emerging epidemiological trends.

Although there are specific recommendations for the air control for the prevention of IFD caused by filamentous fungi [6], air sampling and HEPA filter usage varied notably across institutions. Only about a quarter of institutions routinely conducted air sampling, with lung and heart transplant units showing the highest adoption rates. The use of HEPA filters was more common, especially in lung and heart transplant units, reflecting heightened concern for air quality in these high-risk areas. This variation underscores the need for updated standardized air quality protocols in each center to ensure consistent patient safety across transplant centers. In parallel, it is reasonable to assert that institutions specializing in lung transplantation prioritize air quality, as the lungs are the organs most directly exposed to airborne particulate matter and are essential for efficient blood oxygenation, alongside the heart. Furthermore, IFD can be directly visualized in the bronchial tree, especially at the anastomosis, through bronchoscopy—a minimally invasive procedure that can be easily and rapidly repeated for ongoing assessment. Yet, further analyses are needed to determine whether the disparities in air sampling and HEPA filter implementation stem from a lack of attention at individual hospitals or are tailored to specific local requirements.

The majority of institutions had kidney transplants units. This distribution likely stems from the procedure's technical feasibility, organ availability and global need prevalence [7]. Liver and heart transplants units followed as the next most frequently reported procedures. Lung transplantation units reported more limited data, likely due to the greater complexity associated with these procedures.

Antifungal prophylaxis practices varied significantly among different organ transplantations. While all centers administered antifungal prophylaxis following lung transplantation, the proportion decreased to one-third for kidney transplants. This difference is likely influenced by published epidemiological data, the reported incidences of post-transplant IFD, as well as local epidemiological factors and clinical experience [8]. In the analyzed units, universal prophylaxis was most common in lung transplants, reflecting a heightened perceived IFD risk in these patients. Liver and bowel transplant recipients also showed substantial prophylaxis rates, often triggered by more specific factors such as organ retransplantation or failure. Kidney transplant protocols primarily targeted potential *Candida* spp. colonization as a trigger for prophylaxis initiation. The variability in prophylaxis triggers underscores the lack of standardized protocols across centers, emphasizing the need for consensus guidelines to harmonize practices and enhance patient outcomes.

There was a considerable variation in the choice of preferred antifungal agents among different transplant types, with liposomal amphotericin B, caspofungin, and fluconazole being commonly utilized. This diversity can be attributed to their widespread availability [9–12], broad spectrum of antifungal coverage [13–19], and tissue penetration capabilities [20]. The duration of prophylactic treatment varied significantly between organ types, with most centers adhering to a regimen lasting up to 28 days post-transplantation. Notably, lung transplant protocols often extended prophylaxis beyond 28 days, particularly to oppose IFD from *Aspergillus* spp. This variability underscores the necessity for more precise guidelines and studies regarding the selection and duration of antifungal prophylaxis tailored to specific transplant scenarios and associated risks, including the option antifungal prophylaxis duration. As of 2024, antifungal prophylaxis is advancing with the emergence of new agents such as fosmanopegix, ibrexafungerp, olorofim, opelconazole, and rezafungin. Among these, rezafungin is particularly noteworthy for solid organ transplantation patients due to its effectiveness against *Candida* spp. IFD. This long-acting echinocandin, currently available in select regions, is a key focus because it allows for once-weekly dosing, potentially enhancing adherence to prophylactic regimens. In addition, opelconazole, an inhaled triazole, shows promise for lung transplant recipients due to its activity against *Aspergillus* spp. However, further real-world evidence is essential to determine the optimal applications of these antifungal agents in clinical practice. [21]

The data on estimated breakthrough IFD during the 10 years prior to survey participation indicate significant variation in incidence across transplant types and pathogens. *Candida* spp. IFD were most prevalent in liver transplants, while *Aspergillus* spp. IFD were most common in lung transplants, similar to previous reports [1]. The presence of Mucorales IFD, although less frequent, highlights the importance of maintaining vigilance for these rarer pathogens. The wide range of reported cases underscores the necessity for robust surveillance and tailored prophylaxis strategies to mitigate these IFD effectively.

The findings from this survey highlight several critical considerations. First, there is a pressing need for standardized protocols across transplant centers, focusing on air quality measures, triggers for prophylaxis initiation, selection of antifungal agents, and duration of prophylactic treatment. This standardization is crucial for ensuring consistent and effective management of IFD. Second, given the current variability in IFD incidence and prophylaxis practices, tailored strategies based on specific transplant types and local epidemiology are essential to optimize patient outcomes. Third, enhancing research, including surveillance of IFD and ongoing investigation into the efficacy of different prophylactic regimens, is vital to address the challenges posed by evolving fungal epidemiology and emerging resistant species. Fourth, increasing awareness and providing training for healthcare providers on the latest antifungal prophylaxis practices and guidelines can significantly improve adherence and ultimately enhance patient outcomes. Lastly, it is noteworthy that one in five surveyed centers lacks a dedicated team of infection specialists within the transplant unit. The presence of these specialists is critical for effectively managing the complexities associated with antifungal prophylaxis and infection control in this high-risk patient population. Infection specialists are instrumental in developing and implementing evidence-based guidelines, facilitating timely diagnosis and treatment of IFD, and providing ongoing education to the transplant team regarding the evolving fungal epidemiology and the emergence of resistant species. Their expertise is vital for optimizing prophylactic strategies, improving clinical outcomes, and ensuring that transplant centers maintain vigilance in response to changing IFD patterns [22].

Limitations of the study include several factors that may impact the generalizability and interpretation of the findings. Firstly, the survey relied on voluntary participation from medical institutions, potentially introducing selection bias towards centers more actively engaged in transplant-related research or those with established antifungal prophylaxis protocols. This could limit the representation of practices in smaller or less research-focused institutions. Secondly, the use of an online questionnaire for data collection may have resulted in incomplete or inaccurate responses, including for instance the potential inaccuracy of reported estimated breakthrough IFD rates, which were likely based on participants' personal impressions or general surveillance data rather than a systematic review of center-specific records. Nonetheless, formal monitoring efforts have minimized the potential impact on the accuracy and reliability of the reported data. Thirdly, the lack of statistical comparisons due to the exploratory nature of the analysis limits the ability to draw definitive conclusions about associations or causality between antifungal prophylaxis practices and clinical outcomes. Furthermore, newer antifungal agents such as fosmanogepix, ibrexafungerp, opelconazole or rezafungin were not included into analysis due to their current clinical investigation, current limitation to compassionate use, or restricted accessibility due to regulatory and distribution barriers. In parallel, this study did not address antifungal prophylaxis strategies specifically for multi-organ transplant recipients nor the role and utility of diagnostic fungal tests, which represents an area for future investigation. Finally, the predominance of data from Europe, may not fully capture practices in other regions with different healthcare infrastructures or epidemiological profiles, thus potentially limiting the generalizability of the findings globally.

This international survey reveals the wide variability in antifungal prophylaxis practices across solid organ transplant centers, despite common risks for IFD. The lack of consistent, evidence-based guidelines—along with differences in initiation criteria, prophylactic agents, and duration—emphasizes the pressing need for updated, unified recommendations. These should account for shifting fungal epidemiology, advances in diagnostics, and the emergence of novel antifungal therapies. Our results offer a critical foundation to guide future clinical trials and the creation of comprehensive, population-specific evidence-based guidelines for this vulnerable group.

## Supplementary Information

Below is the link to the electronic supplementary material.

Supplementary file1 (DOCX 17 KB)

## Data Availability

The corresponding author can provide the data supporting the findings of this study upon a reasonable request.
