# Correction: Current trends on antifungal prophylaxis in solid organ transplantation: a study from ESCMID-EFISG, ESCMID-ESGICH, SITA, and SEIMC-GESITRA-IC

**DOI:** 10.1007/s15010-025-02625-6

**Published:** 2025-09-01

**Authors:** Jon Salmanton-García, Alessandro Giacinta, Maddalena Giannella, Antonio Vena, Patricia Muñoz, Oliver A. Cornely, Maricela Valerio

**Affiliations:** 1 Institute of Translational Research, Cologne Excellence Cluster On Cellular Stress Responses in Aging-Associated Diseases (CECAD), Faculty of Medicine, and University Hospital Cologne, University of Cologne, Herderstraße 52, 50931 Cologne, Germany; 2 Department I of Internal Medicine, Center for Integrated Oncology Aachen Bonn Cologne Duesseldorf (CIO ABCD) and Excellence Center for Medical Mycology (ECMM), Faculty of Medicine, University of Cologne, University Hospital Cologne, Cologne, Germany; 3 German Centre for Infection Research (DZIF), Partner Site Bonn-Cologne, Cologne, Germany; 4 Department of Clinical Microbiology and Infectious Diseases, Hospital General Universitario Gregorio Marañón, Madrid, Spain; 5 Division of Infectious Diseases, Department of Medicine, University of Udine, Udine, Italy; 6 Infectious Diseases Unit, IRCCS-Sant’Orsola Polyclinic, Bologna, Italy; 7 Department of Medical and Surgical Sciences, University of Bologna, Bologna, Italy; 8 Department of Health Sciences (DISSAL), University of Genoa, Genoa, Italy; 9 Clinica Malattie Infettive, IRCCS San Martino Polyclinic Hospital, Genoa, Italy; 10 Instituto de Investigación Sanitaria Gregorio Marañón, Madrid, Spain; 11 Department of Medicine, School of Medicine, Universidad Complutense de Madrid, Madrid, Spain; 12 Centro de Investigación Biomédica en Red de Enfermedades Respiratorias (CIBERES), Instituto de Salud Carlos III, Madrid, Spain; 13 Clinical Trials Centre Cologne (ZKS Köln), Faculty of Medicine, University of Cologne, Cologne, Germany

**Correction: Infection** 10.1007/s15010-025-02575-z

In this article Table 2 has been cut after the section “Liver”

**Table 2:**
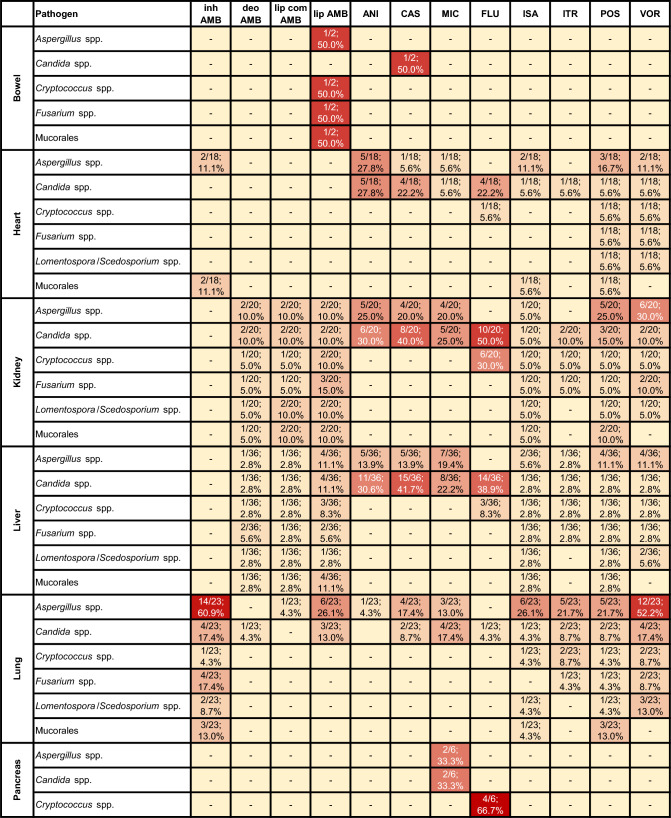
Cross-reference table of administered antifungal drugs for prophylactic use, categorized by pathogen and organ

Denominator corresponds to the number of transplantation units performing prophylaxis for the respective organ. Numerators can be superadditive

Cell colors span from pale yellow to deep red. The intensity of the color indicates the proportionately higher proportional of the respective antifungal in the corresponding organ-pathogen prophylaxis

*ANI* anidulafungi, *CAS* caspofungin, *deo AMB* deoxycholate AMB, *FLU* fluconazole, *inh AMB* inhaled AMB, *ISA* isavuconazole, *ITR* itraconazole, *lip AMB* liposomal AMB, *lip com AMB* lipid complex AMB, *MIC* micafungin, *POS* posaconazole, *VOR* voriconazole, *spp.* species

The author affiliations are given incorrect. They should read: 1. Institute of Translational Research, Cologne Excellence Cluster on Cellular Stress Responses in Aging-Associated Diseases (CECAD), Faculty of Medicine, and University Hospital Cologne, University of Cologne, Herderstraße 52, 50931, Cologne, Germany

Department I of Internal Medicine, Center for Integrated Oncology Aachen Bonn Cologne Duesseldorf (CIO ABCD) and Excellence Center for Medical Mycology (ECMM), University of Cologne, Faculty of Medicine, University Hospital Cologne, Cologne, Germany

German Centre for Infection Research (DZIF), Partner Site Bonn-Cologne, Cologne, Germany

2. Department of Clinical Microbiology and Infectious Diseases, Hospital General Universitario Gregorio Marañón, Madrid, Spain

Division of Infectious Diseases, Department of Medicine, University of Udine, Udine, Italy

3. Infectious Diseases Unit, IRCCS-Sant’Orsola Polyclinic, Bologna, Italy

Department of Medical and Surgical Sciences, University of Bologna, Bologna, Italy

4. Department of Health Sciences (DISSAL), University of Genoa, Genoa, Italy

Clinica Malattie Infettive, IRCCS San Martino Polyclinic Hospital, Genoa, Italy

5. Instituto de Investigación Sanitaria Gregorio Marañón, Madrid, Spain

Department of Medicine, School of Medicine, Universidad Complutense de Madrid, Madrid, Spain

Centro de Investigación Biomédica en Red de Enfermedades Respiratorias (CIBERES), Instituto de Salud Carlos III, Madrid, Spain

Department of Clinical Microbiology and Infectious Diseases, Hospital General Universitario Gregorio Marañón, Madrid, Spain

6. Institute of Translational Research, Cologne Excellence Cluster on Cellular Stress Responses in Aging-Associated Diseases (CECAD), University of Cologne, Faculty of Medicine, and University Hospital Cologne, Herderstraße 52, 50931, Cologne, Germany

Department I of Internal Medicine, Center for Integrated Oncology Aachen Bonn Cologne Duesseldorf (CIO ABCD) and Excellence Center for Medical Mycology (ECMM), University of Cologne, Faculty of Medicine, University Hospital Cologne, Cologne, Germany

German Centre for Infection Research (DZIF), Partner Site Bonn-Cologne, Cologne, Germany

Clinical Trials Centre Cologne (ZKS Köln), University of Cologne, Faculty of Medicine and University Hospital Cologne, Cologne, Germany

7. Instituto de Investigación Sanitaria Gregorio Marañón, Madrid, Spain

Department of Medicine, School of Medicine, Universidad Complutense de Madrid, Madrid, Spain

Centro de Investigación Biomédica en Red de Enfermedades Respiratorias (CIBERES), Instituto de Salud Carlos III, Madrid, Spain

Department of Clinical Microbiology and Infectious Diseases, Hospital General Universitario Gregorio Marañón, Madrid, Spain

The original article has been corrected.

