# Supplementary material for: Current trends on antifungal prophylaxis in solid organ transplantation: a study from ESCMID-EFISG, ESCMID-ESGICH, SITA, and SEIMC-GESITRA-IC
Source: Infection. 2025 Jul 4;53(6):2411–20. doi: 10.1007/s15010-025-02575-z (PMC12675730; doi:10.1007/s15010-025-02575-z)
Supplement: Supplementary file 1 — Supplementary file1 (DOCX 17 KB) [file 15010_2025_2575_MOESM1_ESM.docx]

**SUPPLEMENTARY TABLES**

**Supplementary table 1.** Cross-reference table of antifungal drug administration duration for prophylactic use, categorized by pathogen and organ.

|  |  | **Bowel** | | **Heart** | | **Kidney** | | **Liver** | | **Lung** | | **Pancreas** | |
| --- | --- | --- | --- | --- | --- | --- | --- | --- | --- | --- | --- | --- | --- |
|  |  | n | % | n | % | n | % | n | % | n | % | n | % |
| ***Aspergillus* spp.** | ≤7 days | 0/2 | 0.0 | 0/18 | 0.0 | 1/20 | 5.0 | 1/36 | 2.8 | 0/23 | 0.0 | 0/6 | 0.0 |
|  | 7-13 days | 2/2 | 100.0 | 5/18 | 27.8 | 1/20 | 5.0 | 3/36 | 8.3 | 3/23 | 13.0 | 1/6 | 16.7 |
|  | 14-20 days | 0/2 | 0.0 | 4/18 | 22.2 | 1/20 | 5.0 | 7/36 | 19.4 | 1/23 | 4.3 | 3/6 | 50.0 |
|  | 28 days | 0/2 | 0.0 | 1/18 | 5.6 | 4/20 | 20.0 | 4/36 | 11.1 | 0/23 | 0.0 | 0/6 | 0.0 |
|  | >28 days | 0/2 | 0.0 | 3/18 | 16.7 | 1/20 | 5.0 | 2/36 | 5.6 | 18/23 | 78.3 | 0/6 | 0.0 |
| ***Candida* spp.** | ≤7 days | 1/2 | 50.0 | 1/18 | 5.6 | 5/20 | 25.0 | 6/36 | 16.7 | 2/23 | 8.7 | 3/6 | 50.0 |
|  | 7-13 days | 1/2 | 50.0 | 9/18 | 50.0 | 1/20 | 5.0 | 8/36 | 22.2 | 3/23 | 13.0 | 0/6 | 0.0 |
|  | 14-20 days | 0/2 | 0.0 | 2/18 | 11.1 | 1/20 | 5.0 | 8/36 | 22.2 | 0/23 | 0.0 | 4/6 | 66.7 |
|  | 28 days | 0/2 | 0.0 | 2/18 | 11.1 | 4/20 | 20.0 | 7/36 | 19.4 | 3/23 | 13.0 | 1/6 | 16.7 |
|  | >28 days | 0/2 | 0.0 | 2/18 | 11.1 | 6/20 | 30.0 | 5/36 | 13.9 | 8/23 | 34.8 | 1/6 | 16.7 |
| ***Cryptococcus* spp.** | ≤7 days | 1/2 | 50.0 | 1/18 | 5.6 | 2/20 | 10.0 | 1/36 | 2.8 | 1/23 | 4.3 | 1/6 | 16.7 |
|  | 7-13 days | 1/2 | 50.0 | 0/18 | 0.0 | 0/20 | 0.0 | 0/36 | 0.0 | 0/23 | 0.0 | 0/6 | 0.0 |
|  | 14-20 days | 0/2 | 0.0 | 0/18 | 0.0 | 1/20 | 5.0 | 2/36 | 5.6 | 0/23 | 0.0 | 1/6 | 16.7 |
|  | 28 days | 0/2 | 0.0 | 2/18 | 11.1 | 2/20 | 10.0 | 2/36 | 5.6 | 0/23 | 0.0 | 0/6 | 0.0 |
|  | >28 days | 0/2 | 0.0 | 0/18 | 0.0 | 2/20 | 10.0 | 2/36 | 5.6 | 2/23 | 8.7 | 0/6 | 0.0 |
| ***Fusarium* spp.** | ≤7 days | 0/2 | 0.0 | 0/18 | 0.0 | 0/20 | 0.0 | 0/36 | 0.0 | 0/23 | 0.0 | 1/6 | 16.7 |
|  | 7-13 days | 2/2 | 100.0 | 1/18 | 5.6 | 1/20 | 5.0 | 1/36 | 2.8 | 1/23 | 4.3 | 0/6 | 0.0 |
|  | 14-20 days | 0/2 | 0.0 | 0/18 | 0.0 | 1/20 | 5.0 | 1/36 | 2.8 | 0/23 | 0.0 | 1/6 | 16.7 |
|  | 28 days | 0/2 | 0.0 | 1/18 | 5.6 | 2/20 | 10.0 | 1/36 | 2.8 | 0/23 | 0.0 | 0/6 | 0.0 |
|  | >28 days | 0/2 | 0.0 | 0/18 | 0.0 | 1/20 | 5.0 | 1/36 | 2.8 | 3/23 | 13.0 | 0/6 | 0.0 |
| ***Lomentospora prolificans/***  ***Scedosporium* spp.** | ≤7 days | 0/2 | 0.0 | 0/18 | 0.0 | 0/20 | 0.0 | 0/36 | 0.0 | 0/23 | 0.0 | 0/6 | 0.0 |
|  | 7-13 days | 0/2 | 0.0 | 0/18 | 0.0 | 0/20 | 0.0 | 0/36 | 0.0 | 0/23 | 0.0 | 0/6 | 0.0 |
|  | 14-20 days | 0/2 | 0.0 | 0/18 | 0.0 | 1/20 | 5.0 | 1/36 | 2.8 | 0/23 | 0.0 | 1/6 | 16.7 |
|  | 28 days | 0/2 | 0.0 | 1/18 | 5.6 | 2/20 | 10.0 | 1/36 | 2.8 | 0/23 | 0.0 | 0/6 | 0.0 |
|  | >28 days | 0/2 | 0.0 | 0/18 | 0.0 | 1/20 | 5.0 | 1/36 | 2.8 | 4/23 | 17.4 | 0/6 | 0.0 |
| **Mucorales** | ≤7 days | 1/2 | 50.0 | 1/18 | 5.6 | 1/20 | 5.0 | 1/36 | 2.8 | 1/23 | 4.3 | 1/6 | 16.7 |
|  | 7-13 days | 1/2 | 50.0 | 0/18 | 0.0 | 0/20 | 0.0 | 0/36 | 0.0 | 0/23 | 0.0 | 0/6 | 0.0 |
|  | 14-20 days | 0/2 | 0.0 | 0/18 | 0.0 | 1/20 | 5.0 | 2/36 | 5.6 | 0/23 | 0.0 | 1/6 | 16.7 |
|  | 28 days | 0/2 | 0.0 | 0/18 | 0.0 | 2/20 | 10.0 | 1/36 | 2.8 | 0/23 | 0.0 | 0/6 | 0.0 |
|  | >28 days | 0/2 | 0.0 | 1/18 | 5.6 | 1/20 | 5.0 | 1/36 | 2.8 | 3/23 | 13.0 | 0/6 | 0.0 |

Denominator corresponds to the number of transplantation units performing prophylaxis for the respective organ. Numerators can be superadditive.

spp., species
